# Early Class III Treatment Using a Hybrid Rapid Palatal Expander and Facemask in a Patient with Partially Edentulous Maxilla Post MNTI Removal: A Case Report

**DOI:** 10.3390/healthcare10091746

**Published:** 2022-09-12

**Authors:** Valeria Luzzi, Beatrice Marasca, Marta Mazur, Artnora Ndokaj, Valentina Pirro, Mariana Guaragna, Federica Altieri, Gaetano Ierardo

**Affiliations:** UOC Paediatric Dentistry, Department of Oral and Maxillofacial Sciences, Sapienza University of Rome, 00161 Rome, Italy

**Keywords:** hybrid palatal expander, facemask, melanotic neuroectodermal tumour, orthodontic miniscrew

## Abstract

This case report describes the orthodontic treatment of a 9-year-old girl who presented with multiple agenesis, maxillary contraction, and skeletal Class III malocclusion after the surgical removal of a melanocytic neuroectodermal tumour of infancy (MNTI) or the so-called melanocytic progonoma at 40 days of age. The lack of dental anchorage in the posterior segment of the second quadrant and the search for maximum control during suture expansion to reduce dental effects led to the use of a hybrid rapid palatal expander (RPE) with dental anchorage in the first quadrant and skeletal anchorage on the two miniscrews placed in the second quadrant, to allow a more even distribution of expansion forces. The expansion procedures performed with the hybrid anchorage device and extraoral traction demonstrate the possibility of solving the contraction in the posterior segments and anterior crossbite in a few months with maximum control of the applied forces, despite the objective difficulties related to the specificity of the case.

## 1. Introduction

The treatment of class III malocclusions associated with transverse anomalies of the jaw in a growing patient is one of the most discussed chapters in the orthodontic literature. Class III malocclusions are often associated with unilateral or bilateral cross bite, which is evident when both dental arches are in the usual bite. The etiopathogenesis of this malocclusion is multifactorial. From a clinical point of view, the malocclusion may or may not be in the basal structure and therefore occurs in an alveolar, skeletal, or, more often, mixed form [1].

Early treatment with rapid maxillary expander (RME) and face mask is the effective procedure of choice to restore the sagittal and transverse dimensions of the skeleton in a developing patient by combining orthopaedic and dental effects, based on the biomechanical principle of separating and remodelling the middle palatal suture and intermaxillary sutures.

Rapid palatal expansion with dental anchorage alone can be associated with several side effects:

- Vestibular inclination of the anchorage teeth with eruption of the palatal cusps of the posterior teeth;
- Rotation of the lower jaw clockwise;
- Unstable and relapsing expansion;
- Decrease of the dental-periodontal integrity of anchorage teeth and teeth subjected to expansive forces.

In 2010, Lee et al. proposed a miniscrew-assisted rapid palatal expander (MARPE) to remove undesirable dental–alveolar effects and optimize the ability to expand the skeleton [2].

Seong et al. demonstrated that the force distribution is much better in hybrid devices than in devices using purely dental anchorage [3].

The use of these devices seems to significantly reduce the therapeutic indications for surgically assisted rapid palatal expansion (SARPE) and, consequently, the operational risk associated with this type of surgical procedure [4]. The choice of using hybrid palatal expanders with the introduction of TADS is also adopted in patients of developmental age in cases of fragility of the dental–alveolar apparatus and in cases of agenesis with the resulting reduction in the number of tooth elements used as anchorage. Facial mask therapy applied to miniscrew devices also shows fewer negative effects and a more efficient distribution of forces over the jaw complex compared with face mask therapy with sole dental anchorage [5].

Melanotic neuroectodermal tumour of infancy (MNTI) is rare, rapidly growing, pigmented neoplasm of neural crest origin.

The World Health Organization (WHO) has classified it as a benign congenital anomaly with expansive and aggressive growth, although a few cases of a potentially malignant transformation have been reported. It occurs mainly in the first 6 months of life (82%) and occurs mainly in the upper jaw (68–80%). However, it has also been described in the lower jaw (5.8%), head (10.8%), brain (4.3%) and other parts of the body [6]. This tumour has been previously identified by a variety of names including congenital melanocytic carcinoma, melanocytic progonoma, retinal gyrus tumour, retinal choroid, pigmented adenoma, melanocyte epithelial odontoma, melanoma enamel, and melanoma.

The first case was described by Krompecher in 1918 as congenital melanomacarcinoma [6,7].

There was uncertainty about the origin of the cancer for many years, until in 1966, Borello and Gorlin [8] recognized its origin from nerve crest cells, additionally because many of these patients excrete vanylmandelic acid (VMA) in the urine as found in other neuroectodermal tumours (neuroblastoma, ganglioneuroblastoma, pheochromocytoma). The neural crest cells are totipotent cells that can differentiate into odontogenic ectomesenchyme cells, melanocytes and ganglion neurons.

Macroscopically, the MNTI is a rapidly growing, osteolytic, painless, tense, blue-black neoformation covered with non-ulcerated mucosa. The melanocytic neuroectodermal tumour microscopically has two types of cells: cuboidal epithelial-type cells and smaller, round cells with a dark nucleus and a rare neuroblast-like cytoplasm. The presence of the latter cells necessitates differential diagnosis for Ewing’s sarcoma, embryonic rhabdomyosarcoma, lymphoblastic lymphoma, mesenchymal chondrosarcoma and neuroblastoma.

Surgical resection [7] of neoformation is the treatment of choice and is mostly successful, although local recurrences are reported in 10–15% of cases. Malignant MNTIs are rare, with a metastatic rate of 3% of cases.

The aim of this case report was to describe the orthodontic treatment of a 9-year-old girl who presented with multiple agenesis, maxillary contraction and skeletal Class III malocclusion after the surgical removal of a melanocytic neuroectodermal tumour of infancy (MNTI) or the so-called melanocytic progonoma at 40 days of age.

## 2. Patient Information and Clinical Findings

A 9-year-old female patient came to the operative unit UOC Paediatric Dentistry, in May 2021. The frontal extraoral examination showed a facial asymmetry with soft tissue concavity of the left cheek in comparison with the contralateral side, and at the left labial level, the scarring of the extraoral surgical access resulting from the neonatal surgery was evident. In the lateral view a flat profile with mandibular protrusion, retrognathia of the upper jaw and an increase of the nasolabial angle were observed. 

**Figure 1:**
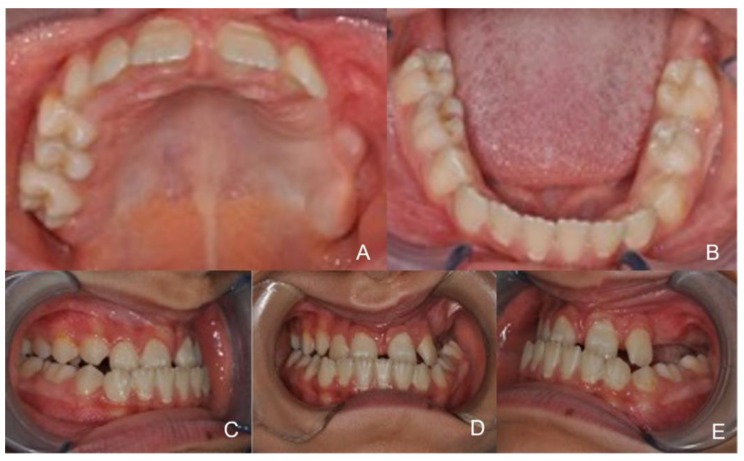
Intraoral view. (**A**): Maxillary occlusal view; (**B**): mandibular occlusal view; (**C**): Right molar relationship; (**D**): frontal view; (**E**): left molar relationship.

**Figure 2:**
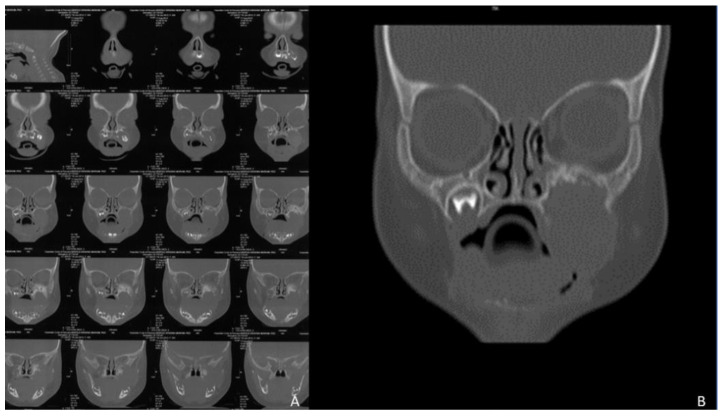
(**A**,**B**): Cone-beam of the maxillofacial region at 40 days of age.

The patient presented a III class malocclusion with an anterior cross bite, a reduced transverse diameter of the maxilla in the posterior sectors, a bilateral cross bite and micrognathia of the upper jaw (Figure 1), in the II quadrant the complete inclusion of 23, 24, and 25 and agenesis of 26 and 27 (Figure 1A) as a result of surgical removal of a melanocytic neuroectodermal tumour of the infancy or the so-called melanocytic progonoma occurred at 40 days of age. Figure 2 A,B shows the CT images of the maxillofacial for the voluminous tumour mass affecting the upper jaw on the left side.

In the here-presented clinical case, the removal of a neoformation from the left upper jaw was performed at the Department of Maxillofacial Surgery at the University of Padua. The neoformation extended widely over much of the left upper jaw, all the way to the lower orbital fissure and the medial fossa of the skull and required removal of both the 26 and 27 tooth germs.

Histological examination of the lesion confirmed the presence of epithelial cells and small round cells arranged in a vesicular aggregation pattern, immersed in the fibrous stroma with the presence of melanic pigment in the cytoplasm, confirming the diagnosis of a melanotic neuroectodermal tumour of infancy.

## 3. Diagnostic Assessment

**Figure 3:**
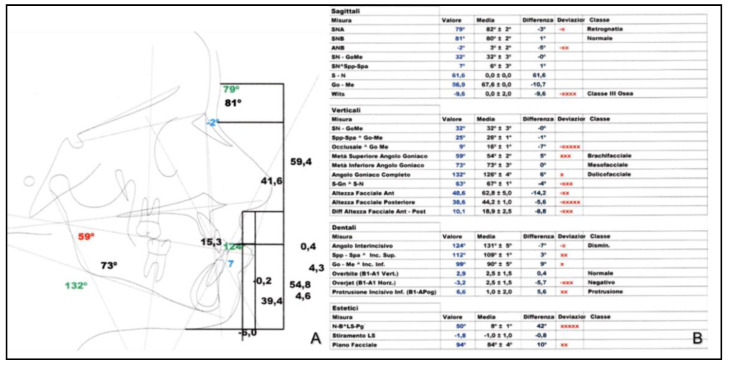
Cephalometric tracing (**A**) and analysis (**B**) before treatment.

Cephalometric analysis (performed with Nemoceph NX) before treatment, revealed class III skeletal malocclusion defect with retrognathic maxilla (ANS 79°), correct mandibular position (SNB 81°) with negative ANB angle (−2°) and Witts index of −9, 6 (Figure 3).

At the dental level, the patient had a Class III molar and canine ratio with a 124° interincisal angle and a negative OVJ of −3.2.

Aesthetically, the patient showed a slight increase in the nasolabial angle and a protruding chin. The patient showed altered cephalometric aesthetic parameters and a tertiary brachiocephalic aesthetic profile.

The lower dental midline was centred with respect to the midline of the face, while the upper was tilted approximately 3mm to the left.

**Figure 4:**
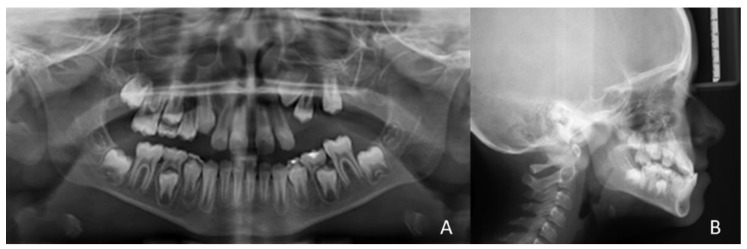
The initial orthopanoramic (**A**) and lateral cephalogram (**B**).

The initial orthopanoramic shows the inclusion of the tooth elements 23, 24, 25 and the absence of 26 and 27 (Figure 4).

**Figure 5:**
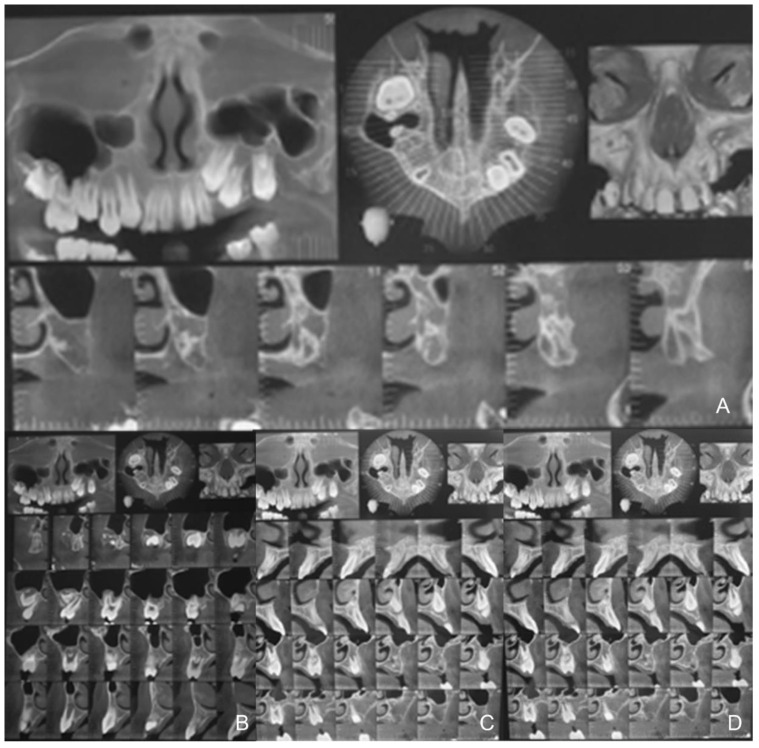
CBCT evaluation of the degree of ossification and miniscrew positioning.

The CBCT evaluation of the degree of ossification (Figure 5) of the middle palatal suture showed a well-presented sutural line along its entire length at high density with no or little interdigitation (Stage A) [9].

### Treatment Objectives

The orthodontic treatment of this first therapeutic phase included the following objectives:

- Correction of bilateral posterior crossbite through orthopaedic expansion procedures avoiding dental compensation.
- Achieving Class I canines and molars relationship.
- Correction of the frontal cross bite.
- Improvement of facial aesthetics.

## 4. Therapeutic Intervention

**Figure 6:**
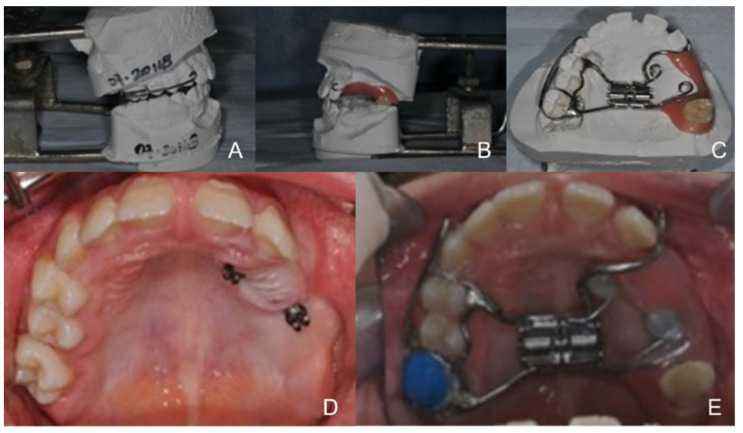
Realization of the hybrid rapid palatal expander with side hooks for the extraoral traction.

The assessment of the type of mesial-palatal suture found in the patient, the lack of dental anchorage in the posterior segment of the second quadrant and the search for maximum control during suture expansion to reduce dental effects led to the use of a hybrid rapid palatal expander with dental anchorage in the first quadrant and skeletal anchorage on the two miniscrews placed in the second quadrant, to allow a more even distribution of expansion forces (Figure 6). The analysis of the CBTC in the axial and transverse projections allowed for an evaluation of the anatomical structures and an easy determination of the sites where the miniscrews should be inserted. In this specific case, it was not considered necessary to use operator-independent insertion methods due to the extent of edentulism in the area identified for the insertion of the miniscrews.

The orthodontic miniscrews (Sweden e Martina, Padua, Italy) made of grade 5 titanium with a self-tapping thread with a diameter of 1.80 mm and a transmucosal neck of 1.30 mm were used. The 8 mm long miniscrews allow an immediate load of 50 to 300 g depending on the planned orthodontic treatment.

**Figure 7:**
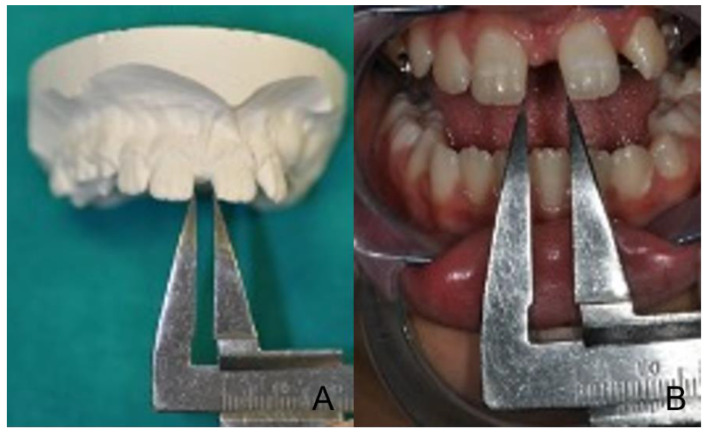
(**A**): Interincisive diastema control at the treatment onset = 4mm; (**B**): After 15 days of activations = 6 mm.

**Figure 8:**
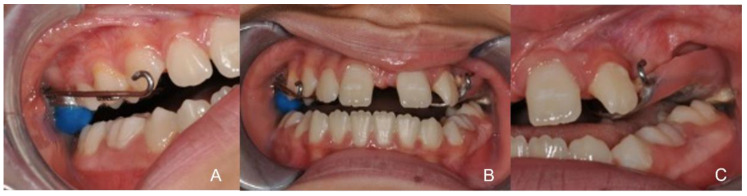
Intraoral images. (**A**) Right lateral view; (**B**) Frontal view; (**C**) Left lateral view.

The treatment protocol included 4 screw activations immediately after insertion, followed by 2 activations daily for a total of 15 days and a predicted expansion of 4 mm (Figure 7 and Figure 8).

**Figure 9:**
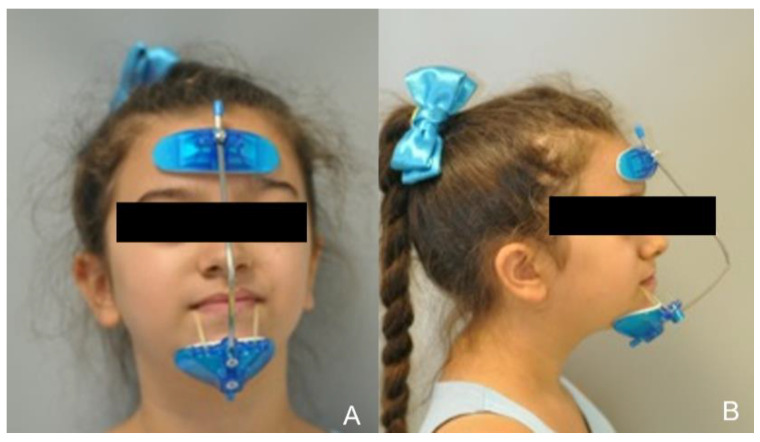
Extraoral traction. (**A**): Frontal view; (**B**): Lateral view.

At the end of the expansion, the device was blocked with a composite resin placed inside the expansion bolt. The extraoral traction was used 20 h per day, for a total of 6 months with 5/16 elastics (Face Mask, Aestetika othodontics, Murster Germany) (Figure 9).

## 5. Outcomes and Follow-Up

**Figure 10:**
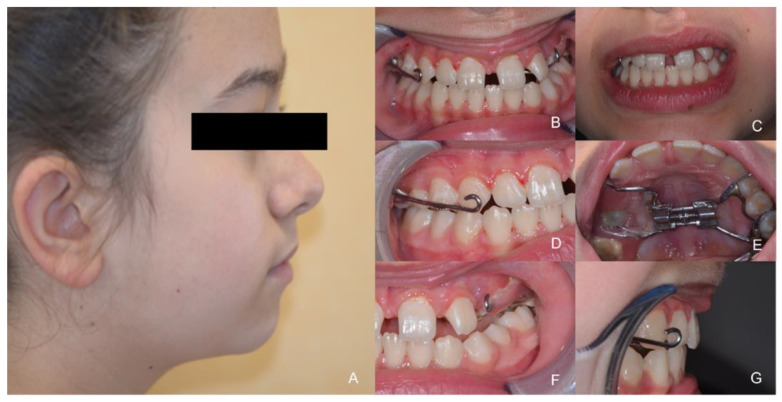
Result after treatment. (**A**) The profile of the patient; (**B**) Frontal intraoral view; (**C**) Frontal extraoral view; (**D**) Right lateral intraoral view; (**E**) Occlusal view; (**F**) Left lateral intraoral view; (**G**) Intraoral profile view.

The expansion procedures performed with the hybrid anchorage device and extraoral traction made it possible to solve the contraction in the posterior segments and anterior crossbite in 8 months of time with maximum control of the applied forces. During the therapy, the patient significantly improved the dental relations of the molars and canines, and the relationship between the arches and the bite improved (Figure 10).

**Figure 11:**
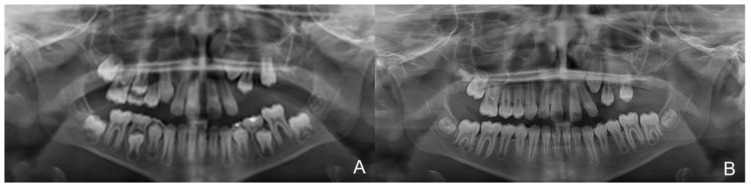
The initial (**A**) and final (**B**) orthopanoramic views.

The Figure 11 shows the orthopanoramic views pre and post treatment.

**Figure 12:**
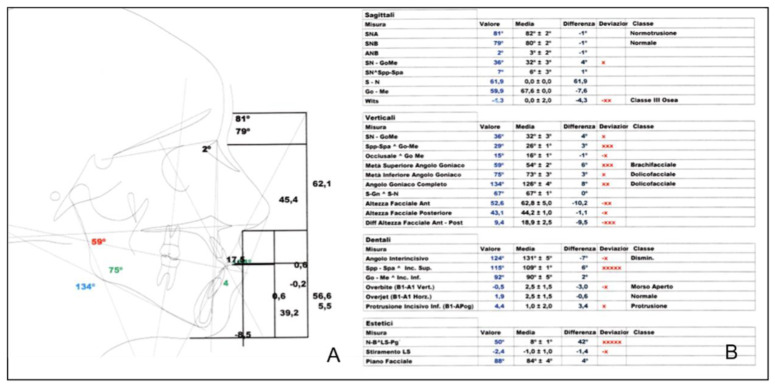
Final cephalometric tracing (**A**) and analysis (**B**).

**Figure 13:**
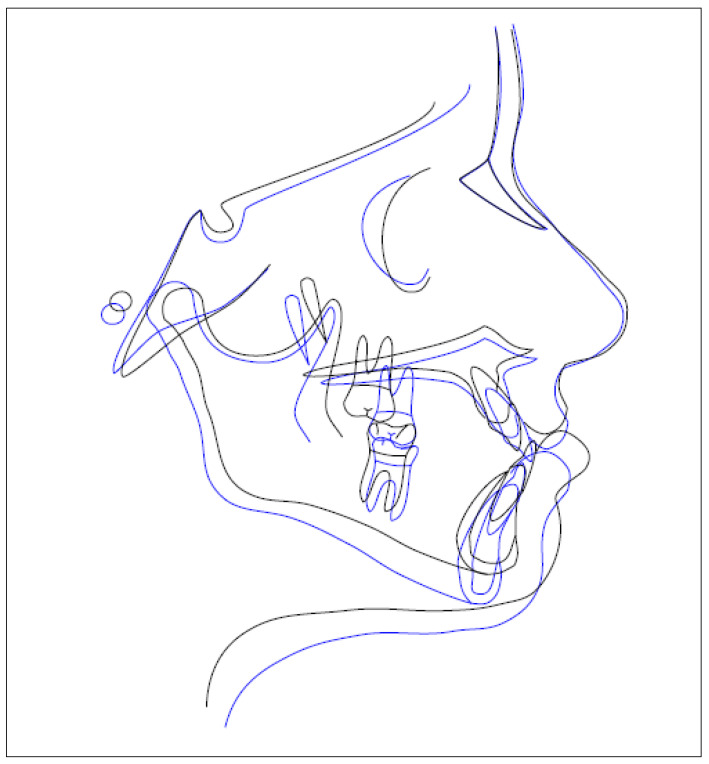
Superimposed pre-treatment (black line) and post-treatment (blue line) cephalometric tracings.

The cephalometric data show normalization of the ANB angle by 2° and SNA and SNB by 81° and 79° respectively, a negative Wits value of −4.3 but halved from the previous value and an improvement in the overjet to 1.9 (Figure 11). The improvement of cephalometric values translates into an improvement of the aesthetic parameters of the face with a more visible harmony of the profile (Figure 12). The Figure 13 shows the profile superimposition before and after treatment.

## 6. Discussion

Correction of contraction of the posterior lateral maxillary and anterior crossbite sectors is most effective when done before the growth spike has occurred [10]. Moreover, it has been documented that maxillary arch advancement before the age of 8 is more effective [11].

The early resolution of a mono- or bilateral crossbite avoids the formation of skeletal defects that can later become structural. For this purpose, the rapid expansion of the palate is certainly one of the most used treatments in orthodontics.

In recent years, miniscrew expanders (TADs) or hybrid expanders with tooth and skeletal anchorage have been increasingly used as an alternative to surgically assisted expansion in adult patients and in late-growth patients. Many authors agree that the use of these devices allows for better control and distribution of applied forces, reducing undesirable dental effects such as dento-alvolar tipping and allowing a more visible maxillary advancement [12].

In the presented case report, a patient in developmental age with a class III malocclusion and partially edentulous was treated with a hybrid palatal expander and protraction facemask.

Orthodontic miniscrew placement was supported by CBCT baseline images without the risk of damaging the anatomical structures due to the lack of tooth elements in the 2nd quadrant. The start of therapy was quick thanks to the possibility of immediate loading. However, even in those cases where the insertion is particularly complicated due to the lack of bone at selected sites, systems have been developed that allow the model to be aligned with CBCT images and create a personalized surgical template.

In the presented case, this first phase of orthodontic treatment made it possible to achieve the goals of skeletal-dental class III correction and aesthetic improvement in a short time, despite the objective difficulties related to the specificity of the case.

In the literature, there are few documented clinical cases of orthodontic treatment in developmental patients who have previously been treated surgically for MNTI; this is mainly due to the rarity of this tumour form [6,13]. For example, the case reported by Hichijo documents the 19-month orthodontic treatment of an anterior crossbite treated with upper jaw expansion in an unoperated patient with a mandibular MNTI who was being monitored due to its subclinical behaviour [14].

More often, extensive implant-prosthetic rehabilitation, the use of obturators in the upper jaw, bone grafts and the use of temporary removable prostheses are documented in both the mandibular and upper jaw after operations to remove the tumour mass. On the other hand, the often locally aggressive behaviour of this form of tumour entails extensive demolition surgery, as in the case documented in this article [15], which requires a multidisciplinary surgical, orthodontic and implant–prosthetic approach given the loss of teeth in the entire third quadrant. 

The most frequent localisation of MNTI in the upper jaw results in the loss of portions of the alveolar process and the maxillary bone with the consequent loss of dental elements and asymmetries of the transverse diameters with consequent soft tissue asymmetries, which are evident clinically in developmental age [16]. Surgery is in all cases, the treatment of first choice, and involves radical resection of the tumour until disease-free tissue margins are obtained. In fact, relapses are associated with non-radical surgery [17].

Recurrences are estimated in the literature to be around 20% and 25% [17]. This kind of recurrence risk, which is high, together with the rapid expansion of the tumour in the first few months of life, necessitates extensive surgical resections with extraoral access routes. This type of surgical approach results in the simultaneous loss of the buds of the permanent teeth and impairment of the growth potentials of the bone structures involved [18,19].

Compared with the cases described in the literature, the present case report shows a more conservative surgical approach, while maintaining the radical character intact. This allowed an orthodontic approach with the objectives of reducing transverse and sagittal deficits in a short time by using hybrid expander and a facial mask. Moreover, the use of modern technology with a minimally invasive clinical approach reflects the emerging era of three-dimensional orthodontics and holistic dentistry [20,21].

The correlation between early tooth loss and reduced upper jaw diameters due to iatrogenic causes, such as the removal of neoformations, cysts, non-syndromic shakes, etc. in early infancy or childhood, is evident. The correlation between hypodontia and reduced maxillary volume is confirmed in cases of cleft lip and palate, underlining the fact that regardless of surgical outcomes, intrinsic factors play a significant role in influencing maxillary growth [22]. In addition, a recent article analysed the morphological differences in the mediopalatine suture in normal versus edentulous situations, showing how tooth loss and functional load considerably affect the morphology of the sutures themselves, in the sense of less vascularisation, reduced sutural width and greater obliteration at the same age [23].

Many studies suggest that MARPE can correct transverse deficits in the upper jaw in young patients while minimising dental effects, constituting a viable alternative to RPE [24,25,26]. Indications for the use of MARPE include conditions of transverse deficits of the upper jaw in conditions of periodontal impairment or reduced number of tooth elements [27].

In the study of Ierardo et al. [28], similar to the present case report, the use of a hybrid rapid palatal expander with mixed anchorage in a case of symmetrical oligodontia of the middle and anterior sectors caused by ectodermal dysplasia was described. In the case we reported, the edentulism involved the entire left upper hemiarchate, with no therapeutic alternatives available.

The clinical timing of the treatment is also an important stage of the therapeutic protocol. In fact, the diastasis of the mid-palatine suture divided into its anterior portion to the incisive foramen or intermaxillary segment, the middle segment from the incisive foramen to the transverse suture of the palatine bone and the posterior segment to the transverse suture of the palatine bone, is obtained in the growing patient by activating the central screw of the REP by 1/2 turn a day for a fortnight. Once the hypercorrection of the cross-bite is achieved, the result is stabilized for 3 months. The results of the palatal expansion are appreciated with a horizontal gain of 4 mm measured at the level of the interincisive diastema. The stability of the results and the absence of dental and periodontal damage are increased with the application of miniscrews (MARPE) to support the palatal expander [29]. Early treatment of Class III has many advantages: It facilitates the eruption of canines and premolars in a normal relationship and eliminates traumatic occlusion of the incisors, which could lead, among other effects, to gingival recession; it provides adequate maxillary growth; and it improves the patient’s personal self-esteem as they develop. In addition, it is described in the literature that Class III discrepancy worsens with age, with growth of the maxilla ending earlier than that of the mandible. After the rapid palatal expansion phase, Class III correction protocols involve the application of the facial mask to initiate maxillary advancement using 5/16-inch elastics with an applied force of 200 g for the first week and then 500 g for 20 h per day for 6 months [30].

## 7. Conclusions

The management of this clinical case has demonstrated the versatility of hybrid anchored palatal expansion devices to treat and resolve complex cases of maxillary contraction and severe edentulous extended to the posterolateral sectors of the maxillary arch, combined with a severe III class skeletal malocclusion. Moreover, the possibility of using new 3D technologies makes these procedures reliable and accessible even to those with modest surgical experience, as they are operator-independent procedures.

## 8. Patient Perspective

The patient was provided essential information for unfamiliar events for better understanding and optimizing patient care.

The patient in this case report did not complain of pain and said she felt comfortable. Pain prevention and control during and after the procedures were essential for nurturing the patient’s relationship with the practitioner, creating confidence, alleviating fear and anxiety, promoting positive behaviour and offering comfort to the patient and, consequently, to her parents.

The patient recovered well postoperatively.

## Data Availability

Not applicable.
